# Lipid remodelling is a widespread strategy in marine heterotrophic bacteria upon phosphorus deficiency

**DOI:** 10.1038/ismej.2015.172

**Published:** 2015-11-13

**Authors:** Marta Sebastián, Alastair F Smith, José M González, Helen F Fredricks, Benjamin Van Mooy, Michal Koblížek, Joost Brandsma, Grielof Koster, Mireia Mestre, Behzad Mostajir, Paraskevi Pitta, Anthony D Postle, Pablo Sánchez, Josep M Gasol, David J Scanlan, Yin Chen

**Affiliations:** 1 Departament de Biologia Marina i Oceanografia, Institut de Ciències del Mar, CSIC, Barcelona, Spain; 2 School of Life Sciences, University of Warwick, Coventry, UK; 3 Department of Microbiology, University of La Laguna, La Laguna, Spain; 4 Department of Marine Chemistry and Geochemistry, Woods Hole Oceanographic Institution, Woods Hole, MA, USA; 5 Institute of Microbiology, Center Algatech, Třeboň, Czech Republic; 6 Clinical and Experimental Sciences, Faculty of Medicine, University of Southampton, Southampton, UK; 7 Center of Marine Biodiversity, Exploitation and Conservation (MARBEC), UMR 9190, CNRS – Université de Montpellier – IRD – IFREMER, Place Eugène Bataillon, Université de Montpellier, Case 93, Montpellier, France; 8 Hellenic Centre for Marine Research, Oceanography Institute, Heraklion, Greece

## Abstract

Upon phosphorus (P) deficiency, marine phytoplankton reduce their requirements for P by replacing membrane phospholipids with alternative non-phosphorus lipids. It was very recently demonstrated that a SAR11 isolate also shares this capability when phosphate starved in culture. Yet, the extent to which this process occurs in other marine heterotrophic bacteria and in the natural environment is unknown. Here, we demonstrate that the substitution of membrane phospholipids for a variety of non-phosphorus lipids is a conserved response to P deficiency among phylogenetically diverse marine heterotrophic bacteria, including members of the *Alphaproteobacteria* and *Flavobacteria*. By deletion mutagenesis and complementation in the model marine bacterium *Phaeobacter* sp. MED193 and heterologous expression in recombinant *Escherichia coli*, we confirm the roles of a phospholipase C (PlcP) and a glycosyltransferase in lipid remodelling. Analyses of the Global Ocean Sampling and *Tara* Oceans metagenome data sets demonstrate that PlcP is particularly abundant in areas characterized by low phosphate concentrations. Furthermore, we show that lipid remodelling occurs seasonally and responds to changing nutrient conditions in natural microbial communities from the Mediterranean Sea. Together, our results point to the key role of lipid substitution as an adaptive strategy enabling heterotrophic bacteria to thrive in the vast P-depleted areas of the ocean.

## Introduction

Phosphorus (P) is required by all living organisms (Karl, 2014). In the oceans, P availability appears to exert a strong selective pressure on organisms growing in low P regions, driving ecotypic divergence in bacterioplankton populations (Coleman and Chisholm, 2010; Grote *et al.*, 2012). The bioavailable P concentration is often low enough to limit phytoplankton (Moore *et al.*, 2013) and heterotrophic bacteria production (Sebastián and Gasol, 2013). When P concentrations are low, heterotrophic bacteria appear to compete with phytoplankton for both organic and inorganic P sources (Havskum *et al.*, 2003; Zubkov *et al.*, 2007). The outcome of this struggle has potential implications for ecosystem productivity, as well as the balance between organic carbon consumption and sequestration (Havskum *et al.*, 2003).

When P is the limiting nutrient, the competitive ability of plankton groups should be inversely proportional to their cellular P quotas (Thingstad and Rassoulzadegan, 1999). Indeed, the imprint of this pressure for cost minimization is shown by the prevalence of streamlined genomes in diverse free-living bacterioplankton in the oligotrophic surface ocean (Swan *et al.*, 2013), noticeably the SAR11 clade representative *Pelagibacter ubique* (Giovannoni *et al.*, 2014) and the cyanobacterium *Prochlorococcus* (Swan *et al.*, 2013). Other than in nucleic acids, the major reservoir for organic P is in membrane phospholipids, which typically account for ~20% of the P content in heterotrophic bacteria and around 10% in some phytoplankton (Van Mooy *et al.*, 2008). Marine phytoplankton have evolved an adaptive strategy to minimize the P cost of their membranes under low phosphate availability by replacing phospholipids with membrane lipids devoid of P (Van Mooy *et al.*, 2006 and 2009). Until recently, abundant marine heterotrophic bacteria were thought to lack the capacity for lipid remodelling in response to P deficiency (Van Mooy *et al.*, 2009) and the view that non-P lipids are primarily derived from phytoplankton has influenced the interpretation of some studies (Close *et al.*, 2014; Gašparović *et al.*, 2014). This view has since been challenged by the observation of glycolipids in the membranes of heterotrophic bacteria from the P-depleted Sargasso Sea (Popendorf *et al.*, 2011) as well as from an isolate of the SAR11 clade, which was originally isolated from P-depleted seawater (Carini *et al.*, 2015). However, it remains unclear whether lipid remodelling is a peculiarity of certain strains of the oligotrophic SAR11 clade or a more general adaptation among marine heterotrophic bacteria to growth in a variety of low P environments.

In this study, we demonstrate that the capacity to remodel lipid content under low P availability is widespread among phylogenetically diverse marine heterotrophic bacteria. We confirm the identity of key genes involved in this process. By deletion mutagenesis and complementation in the model marine heterotrophic bacterium *Phaeobacter* sp. MED193, we conclusively demonstrate that a recently identified phospholipase, PlcP (Zavaleta-Pastor *et al.*, 2010) is central for lipid remodelling. Moreover, analyses of the Global Ocean Sampling (GOS) and *Tara* Oceans metagenome data sets reveal that PlcP is more abundant in surface waters of P-depleted areas of the ocean, such as the Mediterranean Sea and the North Atlantic Ocean. We find that, in addition to its presence in some members of the SAR11 clade bacteria, PlcP is widely distributed across a number of abundant clades of marine bacteria with divergent life strategies, including other *Alphaproteobacteria* (for example, the SAR116 clade, and the marine *Roseobacter* clade), *Gammaproteobacteria* (for example, the SAR86 clade), *Flavobacteria* and *Verrucomicrobia*. By heterologous expression, we validate the activity of a SAR11 glycosyltransferase in the synthesis of glycolipids. Finally, we demonstrate for the first time that lipid remodelling occurs in nature in P-starved heterotrophic bacterial communities, which synthesize a wide array of non-P lipids, some of which were previously ascribed to phytoplankton.

## Materials and methods

### Phosphorus-starvation experiments with marine heterotrophic bacterial isolates

Growth conditions for marine heterotrophic bacteria under P-replete and deplete media are detailed in the Supplementary Materials and Methods. Reverse transcription–PCR (RT-PCR) experiments were performed as described elsewhere (Sebastián and Ammerman, 2009). RT-PCR primers are listed in Supplementary Table S3.

### Searches for PlcP homologues

PlcP homologue sequences were retrieved from marine bacterial isolates using the Integrated Microbial Genomes database and marine metagenomes of the GOS (Rusch *et al.*, 2007) and the *Tara* Oceans (Sunagawa *et al.*, 2015) data sets, using PlcP (MED193_17359) of strain MED193 as a query (*e*-value<10^−40^). Details of the BLAST searches and the phylogenetic analysis can be found in Supplementary Materials and Methods. To search for genes involved in non-P lipid synthesis in the vicinity of *plcP*, the nucleotide sequences 5 kb upstream and downstream of PlcP in the genomes of the marine isolates were extracted. tBLASTn searches on these sequences were performed using characterized non-P lipid synthesis genes as queries (*e*-value<10^−20^; see Supplementary Table S2).

### Construction and complementation of the *plcP* mutant in *Phaeobacter* sp. MED193 and heterologous expression of a SAR11 glycosyltransferase

Construction of a *plc*P deletion mutant of *Phaeobacter* sp. MED193 (MED193_17359) and complementation of the mutant with the native PlcP and SAR11 HTCC7211 PlcP (PB7211_983) were performed following Lidbury *et al.* (2014) and are described in Supplementary Materials and Methods. To characterize the SAR11 HTCC7211 glycosyltransferase, the *agt* homologue (PB7211_960) was chemically synthesized and codon optimized for overexpression in *Escherichia coli*. Glycolipids in recombinant *E. coli* were characterized by mass spectrometry as described in Supplementary Materials and Methods. Bacterial strains, plasmids and the primers used can be found in Supplementary Tables S4 and S5.

### Collection of samples for intact polar diacylglycerolipids (IP-DAGs) in the environment

Samples from the LAMP2011 mesocosm experiment in the Eastern Mediterranean Sea (see Supplementary Material and Methods for details) were size-fractionated through 0.8-μm pore size filters to remove *Synechococcus* and picoeukaryote cells. Microbial communities were then collected onto 0.2-μm pore size filters. Flow cytometry analyses showed that the abundance of *Prochlorococcus* in the samples was negligible. Membrane lipids were extracted and quantified as detailed previously (Popendorf *et al.*, 2013), with the exception of the identification and characterization of glucuronic acid diacylglycerol (GADG) (see below). The bacterial community composition was evaluated by catalyzed reporter deposition fluorescence *in situ* hybridization as described elsewhere (Sebastián *et al.*, 2012).

Natural seawater samples were also collected at the Blanes Bay Microbial Observatory (Western Mediterranean Sea) in August and September 2012 and processed as described above. Microbial community structure was investigated by pyrosequencing of bacterial 16S rRNA genes. Further details on this section can be found in Supplementary Materials and Methods.

### Intact polar lipids measurement

Membrane lipids were analyzed using a high performance liquid chromatography–electrospray ionization–triplequadrupole mass spectrometry (HPLC-ESI-TQMS) method following the protocol detailed in Popendorf *et al.* (2013). Concentrations of GADG were obtained by HPLC/ESI–ion-trap MS (HPLC/ESI-IT-MS) as described in Sturt *et al.* (2004) and modified from Van Mooy and Fredricks (2010). Briefly, samples were injected onto a PrincetonSPHER (Princeton Chromatography Inc., Cranbury, NJ, USA) diol column (2.1 × 150 mm^2^) operating in normal phase mode with a gradient from 100% A to 50% B at 20 min, then to 75% B at 25 min, hold at 75% B for 10 min and then back to 100% A. Flow rate for the gradient was 0.4 ml min^−1^ and then the column was equilibrated with 100% A for 15 min at 1 ml min^−1^. Eluent A was 80:20:0.1:0.04 of n-hexane: 2-propanol: formic acid: 14.8 n ammonium (aq) and eluent B was 90:10:0.1:0.04 of 2-propanol: water: formic acid: 14.8 n ammonium (aq). The electrospray source and the mass spectrometer (Thermo Scientific, Waltham, MA, USA) were configured as previously described (Sturt *et al.*, 2004) and programmed such that the base peak from alternating positive and negative ion full scans (250–2000 Da) was fragmented up to MS^2^.

Molecular ions of *m*/*z* 760, 786, 788 and 814 that eluted at 14.7 min were identified as GADG (Supplementary Figure S4). The major peaks of the MS^2^ spectrum of this group are typical of glycolipids such as monoglycosyl diacylglycerol (MGDG) but showed distinct headgroup neutral losses of *m*/*z* 193 and 211, which are *m*/*z* 14 greater than MGDG. The retention time of GADG was considerably longer than that of MGDG (8.3 min), indicating that it was more polar; this rules out the *m*/*z* 14 difference being due to an additional CH_2_ group. An addition of an N atom is also unlikely because the positive and negative ion molecular ions showed clear evidence of ammonium (+) and formate (−) adducts, which would not occur if there was an N atom in the headgroup (Sturt *et al.*, 2004). This leaves the addition of a carboxylic acid oxygen atom as the remaining possibility, which is indeed consistent with a glucuronic acid headgroup moiety and an assignment of the lipid as GADG. As GADG lipids are not readily commercially available, their molecular ions were quantified against an MGDG calibration curve. Quantities were then normalized to internal standard DNP-PE (1,2-dipalmitoyl-sn-glycero-3-phosphoethanolamine-*N*-(2,4-dinitrophenyl)), which was added before extraction.

## Results

### *plcP* is expressed under P-deficiency conditions in marine heterotrophic bacteria and is essential for lipid remodelling

**Figure 1:**
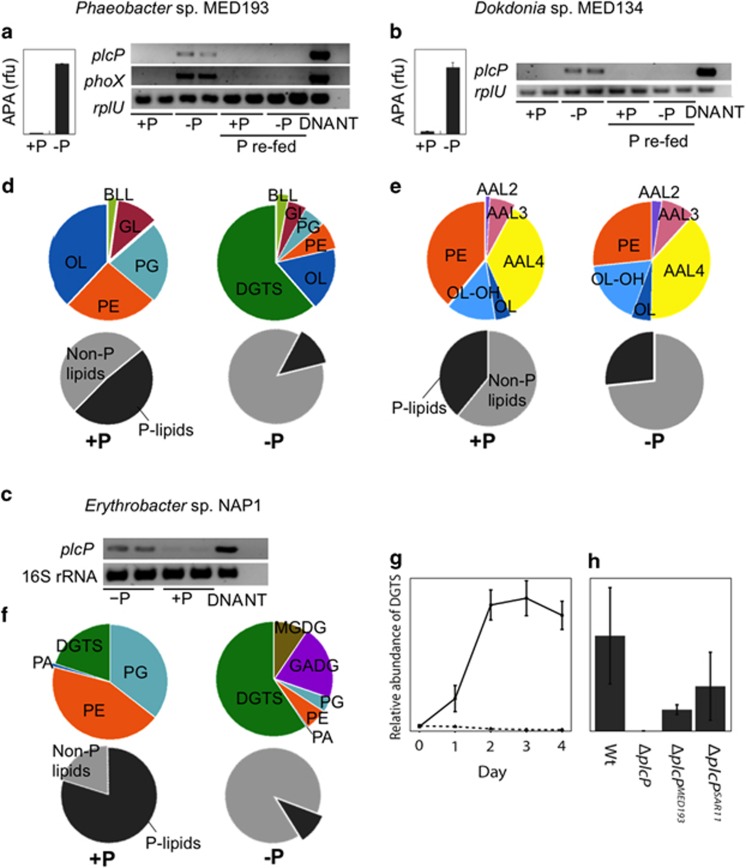
Phosphorus starvation induces alkaline phosphatase activity, *plcP* expression (**a**–**c**) and membrane lipid remodelling (**d**–**f**) in representative marine bacterial heterotrophs. RT-PCR results show *plcP* is transcribed under P-deplete conditions and repressed shortly (2 h) after P is added back to the media (P re-fed). *rplU* or 16S rRNA transcript levels were used as control for cDNA synthesis. Line graph (**g**) shows the accumulation of DGTS in *Phaeobacter* sp. MED193 over time in P-deplete (solid line) but not in P-replete (dashed line) media. DGTS synthesis is observed in wild type (Wt) but not in a *plcP* deletion mutant (Δ*plcP*) (**h**). Complementation with *plcP* from MED193 (Δ*plcP*^*MED193*^) or from SAR11 (Δ*plcP*^*SAR11*^) is sufficient to restore DGTS synthesis. Error bars represent the s.d. of three independent replicates. *phoX*: Alkaline phosphatase PhoX gene, DNA: PCR positive control, NT: no template control, APA: alkaline phosphatase activity, PA: phosphatidic acid, OL: ornithine lipid, GL: glutamine lipid, BLL: an uncharacterized betaine lipid, AAL2, AAL3, AAL4: uncharacterized aminolipids, OL-OH: a hydroxylated ornithine lipid.

To test the hypothesis that phylogenetically diverse marine heterotrophic bacteria synthesize non-P lipids in response to P deficiency, we performed experiments with marine bacterial isolates whose genomes contain homologues to the newly discovered *plcP* from a terrestrial rhizobium (Zavaleta-Pastor *et al.*, 2010). We used the photoheterotrophic alphaproteobacterium *Erythrobacter* sp. NAP1 and two isolates from the Mediterranean Sea, the alphaproteobacterium *Phaeobacter* sp. MED193, which belongs to the *Roseobacter* clade, and the flavobacterium *Dokdonia* sp. MED134. These isolates harbour *plcP* genes sharing 50, 57 and 35% similarity with the rhizobial *plcP*, respectively. We subjected each strain to abrupt P starvation and studied the P starvation response. Alkaline phosphatase activity was used as a diagnostic for P deficiency (Thingstad and Mantoura, 2005) for the two Mediterranean strains, and activity was indeed induced in the P-starved cultures (Figures 1a and b). To investigate whether *plcP* was expressed following P limitation, we used RT-PCR to detect *plcP* transcripts. Expression of *plcP* was induced following P starvation and repressed when P was added back to the culture (Figures 1a–c), showing *plcP* is regulated by P availability.

Each of the strains synthesized a mixture of phospholipids and non-P lipids under both P-replete and P-deplete conditions (Figures 1d–f). Phospholipids detected were phosphatidylethanolamine (PE) and phosphatidylglycerol (PG), although the latter was absent in *Dokdonia* sp. MED134. Non-P lipids were diverse and differed between the three strains. In each case, the proportion of phospholipids decreased following P starvation, whereas non-P lipids became more abundant. Absolute amounts of phospholipids per cell were also determined for the Mediterranean isolates. In *Phaeobacter* sp. MED193, the phospholipid content decreased by half in response to P starvation, contributing 0.27±0.15 × 10^6^ P atoms cell^−1^ compared with 0.54±0.19 × 10^6^ P atoms cell^−1^ when P was not limiting (Supplementary Table S1). A similar response was observed in *Dokdonia* sp. MED134, with phospholipids reduced by around a third following P starvation, from 2.63±1.0 × 10^6^ P atoms cell^−1^ to 1.72±0.28 × 10^6^ P atoms cell^−1^ (Supplementary Table S1).

Different sets of non-P lipids became enriched following P starvation in each of the three isolates. The betaine lipid diacylglyceryl trimethylhomoserine (DGTS) accumulated as the major lipid in *Phaeobacter* sp. MED193 (Figure 1d and g). DGTS also increased in abundance in the membrane of *Erythrobacter* sp. NAP1 when P was limiting, along with two glycolipids, MGDG and GADG (Figure 1f). Compared with the two alphaproteobacterial strains, the flavobacterium *Dokdonia* sp. MED134 exhibited a distinctive membrane lipid composition comprised of a number of different aminolipids (Figure 1e).

To determine whether PlcP is directly involved in lipid remodelling in *Phaeobacter* sp. MED193, we constructed a deletion mutant, Δ*plcP*. When grown under P-limiting conditions, the betaine lipid DGTS was not detectable in the mutant (Figure 1h). To eliminate the possibility of polar effects during mutant construction, we also verified that DGTS synthesis was restored by complementation of the Δ*plcP* mutant with the native *plcP* of *Phaeobacter* sp. MED193 (Δ*plcP*^MED193^). Furthermore, complementation of the Δ*plcP Phaeobacter* sp. MED193 mutant with the *plcP* of the SAR11 HTCC7211 strain (Δ*plcP*^*SAR11*^, Figure 1h) also restored the synthesis of DGTS, indicating that both enzymes have a similar functional behaviour.

### PlcP is abundant and widely distributed among diverse phyla and also expressed in the marine environment

**Figure 2:**
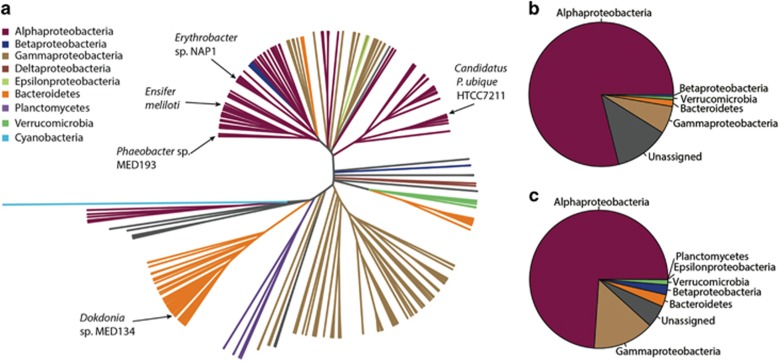
Distribution of PlcP in marine bacteria. (**a**) Phylogenetic relationships of PlcP homologues in marine bacterial isolates and all non-redundant PlcP sequences in the Global Ocean Sampling data set. Sequences were retrieved by BLASTP using *Phaeobacter* sp. MED193 PlcP (MED193_17359) as query (*e*-value<10^−40^). Uncolored sequences are metagenome sequences that did not cluster alongside any isolate sequences. Right panel: Percentage of distribution of metagenome hits within each phylogenetic group in the Global Ocean Sampling (**b**) and *Tara* Oceans (**c**) data sets.

Having established that PlcP is central to the lipid remodelling response to P deficiency, we proceeded to investigate the abundance and distribution of PlcP in the marine environment. We mined the genomes of marine isolates in the Integrated Microbial Genomes database as well as the GOS (Rusch *et al.*, 2007) and *Tara* Oceans metagenomes (Sunagawa *et al.*, 2015) for PlcP homologues. BLAST searches retrieved 216 PlcP homologues in marine isolates (Supplementary Table S2) and >1100 unique environmental PlcP homologues in the GOS metagenomes. PlcP was also found to be ubiquitous in the *Tara* Oceans metagenomes. Phylogenetic analysis of these sequences provided evidence that PlcP is widespread among diverse bacterial phyla, including *Alphaproteobacteria*, *Gammaproteobacteria* and *Flavobacteria* (Figure 2a). Notably, PlcP is present in the two tropical ocean representatives of the ubiquitous SAR11 clade ('*Candidatus* Pelagibacter ubique' sp. HTCC7211 and HTCC7217), as has been recently shown by Carini *et al.* (2015). *Alphaproteobacteria* appeared to dominate the GOS and *Tara* Oceans data sets, accounting for around 75% of the hits (Figures 2b and c). Close relatives of the SAR11 clade PlcP, represented by strain HTCC7211, accounted for 54% and 30% of the hits in the GOS and *Tara* Oceans data sets, respectively. We also found PlcP homologues in single amplified genomes from a number of other abundant lineages of marine bacteria. These included the SAR116 clade of *Alphaproteobacteria*, the gammaproteobacterial SAR86, a flavobacterium and a verrucomicrobium (Dupont *et al.*, 2012; Swan *et al.*, 2013). As an adaptation to P deficiency, PlcP is expected to be more abundant in areas of the oceans where low P availability exerts a strong selective pressure. Analyses of the relative abundance of PlcP in the metagenomes from the GOS and the *Tara* Oceans expeditions showed that PlcP is indeed more abundant in the Mediterranean Sea and the North Atlantic Ocean (Supplementary Figure S1), which are characterized by low phosphate concentrations and high N/P ratios compared with other areas of the oceans (Fanning, 1992; Krom *et al.*, 2010; Wu, 2000). We also analyzed marine metatranscriptomic data sets and found that *plcP* is being transcribed in the marine environment by heterotrophic bacteria belonging to different phylogenetic groups (Supplementary Figure S2).

### Genomic context of *plcP*

**Figure 3:**
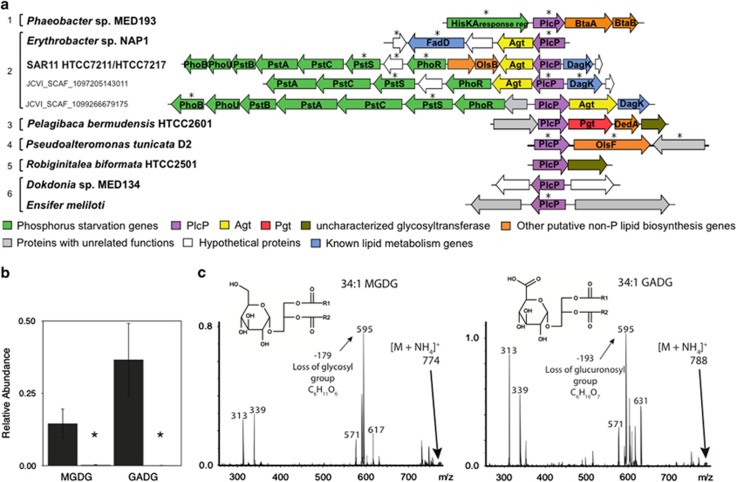
PlcP often appears in an operon with a glycosyltransferase. (**a**) Genomic context of *plcP*. Numbers denote the six types of *plcP* genomic neighbourhood in representative marine heterotrophic bacteria (see Supplemantary Table S2 for further information). Asterisks indicate those genes with an upstream PhoB-binding site (Pho box) and therefore putatively expressed upon phosphorus deficiency. (**b**) Heterologous expression of the putative glycosyltransferase *agt* from SAR11 HTCC7211 results in the accumulation of two glycolipids: MGDG and GADG (dark grey bars) but not in the control harbouring an empty expression plasmid (light grey bars). Abundance is expressed as the peak area of glycolipid relative to that of a phosphatidylcholine internal standard. (**c**) Fragmentation spectra for representative MGDG and GADG species obtained from recombinant *E. coli* harbouring the HTCC7211 *agt* homologue. The two species differ in the neutral loss corresponding to the polar head group (179 and 193 *m/z* for the loss of hexosyl and hexuronic acid groups, respectively). In each case, this loss yields diacylglycerol (595 *m/z*). Two peaks corresponding to monoacylglycerol with 18:1 and 16:0 fatty acids (339 and 313 *m/z*, respectively) are also present.

In order to shed further light on the role of PlcP, we analyzed its genomic context in marine isolates in the Integrated Microbial Genomes data set and environmental metagenomic scaffolds of the GOS data set (Figure 3). Genes involved in the phosphorus starvation response (Pho regulon) are regulated by the two-component system PhoR/PhoB and share a consensus sequence named Pho box in their promoter regions. Using bioinformatics approaches, we identified putative Pho-box binding sites in the PlcP gene clusters. Based on the genomic context of *plcP*, there are broadly six types of gene arrangements, and we detected a gene putatively involved in non-P lipid synthesis in the neighbourhood of *plcP* in around two-thirds of the strains investigated (Supplementary Table S2).

Type 1 is present in several strains, including *Phaeobacter* sp. MED193, in which the two genes necessary for the synthesis of the betaine lipid DGTS, *btaA* and *btaB* (Riekhof *et al.*, 2005), are found downstream of *plcP*. This is consistent with the lipid results obtained in strain MED193 cultures and its mutants (Figures 1d, g and h). The most commonly observed gene in the vicinity of *plcP* (type 2, present in >25% of the bacterial isolates analyzed, Supplementary Table S2) was a putative glycosyltransferase, homologous to the promiscuous glycosyltransferase *agt* just recently identified in *Agrobacterium tumefaciens* (Semeniuk *et al.*, 2014). Strains with *agt* homologues downstream of *plcP* include the environmentally relevant '*Candidatus* Pelagibacter ubique' sp. HTCC7211 and HTCC7217 and *Erythrobacter* sp. NAP1 (Figure 3a). In *A. tumefaciens*, this gene was found to be required for the synthesis of GADG and MGDG under P deficiency (Semeniuk *et al.*, 2014), which is consistent with our finding of those lipids in *Erythrobacter* sp. NAP1 when grown in P-limiting conditions (Figure 1f) and the recent findings of Carini *et al.* (2015). To confirm the role of the glycosyltransferase *agt* in the synthesis of these lipids, we chemically synthesized the gene (PB7211_960) from '*Candidatus* Pelagibacter ubique' sp. HTCC7211 and expressed it in *E. coli*. This resulted in the accumulation of MGDG and GADG (Figures 3b and c), which were not observed in lipid extracts from the same strain transformed with an empty vector (Figure 3b), confirming that this *agt* homologue in HTCC7211 is sufficient and responsible for the synthesis of the two glycolipids MGDG and GADG.

Types 3 and 5 include a number of marine heterotrophic bacteria where *plcP* is located immediately upstream of some putative glycosyltransferases, one of which has been confirmed to be responsible for the synthesis of di- and tri-glycosyldiacylglycerols (Devers *et al.*, 2011). However, in types 4 and 6, *plcP* does not seem to form an operon with its neighbouring genes although genes predicted to be involved in the synthesis of non-P lipids (for example, *btaBA*) are, in many cases, found elsewhere in their genomes (Supplementary Table S2). Together, our analyses of the genomic context of *plcP* support its role in the synthesis of non-P glycolipids and betaine lipids in marine heterotrophic bacteria (Supplementary Figure S3).

### Membrane lipid remodelling in environmental samples

**Figure 4:**
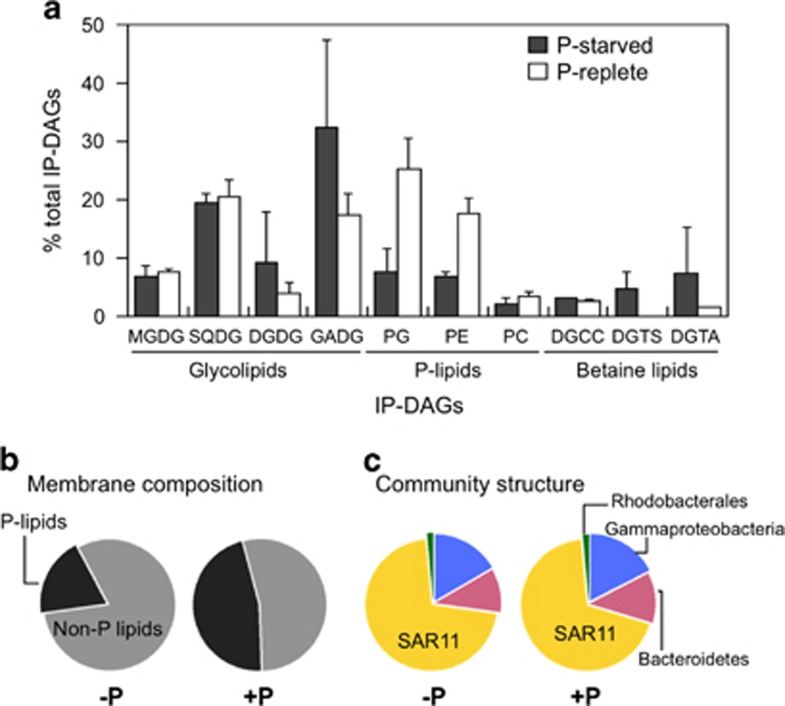
Polar lipids from the heterotrophic bacterial community (0.2–0.8-μm size fraction) in the Eastern Mediterranean during the mesocosm experiment. (**a**) Contribution of IP-DAGs to the membrane composition in the P-starved (control) and P-replete treatments. (**b**) Percentage of contribution of non-P versus P-lipids. (**c**) Bacterioplankton community structure at the time of sampling. PC: phosphatidylcholine, DGCC: diacylglycerylcarboxy-*N*-hydroxymethyl-choline.

To assess whether natural marine heterotrophic bacterial communities indeed replace phospholipids with a variety of non-P lipids upon P deficiency, we analyzed their membrane lipid composition using a size-fractionation approach. We first performed a mesocosm experiment with waters from the Eastern Mediterranean Sea, which is considered one of the most P-starved systems on Earth (Tanaka *et al.*, 2007). Non-P lipids represented >80% of the membrane lipids of heterotrophic bacteria in this system (Figures 4a and b). Four mesocosms were set up, two of which were enriched with phosphate (100 nm), while the other two were left un-amended, as controls. Six days after enrichment, phospholipids had greatly increased in proportion, to represent almost half of the total lipids, a 2–5-fold increase in the membrane lipid P content relative to P-starved controls (Figure 4b, Supplementary Table S1). Despite this shift in the lipid profile, there was little variation in bacterial community composition between treatments at the broad phylogenetic scale (Figure 4c). Interestingly, the most abundant non-P lipid in the P-starved mesocosm was GADG (fourfold higher compared with the P-enriched treatments) (Figure 4a), which has not been reported in marine surface waters previously (Popendorf *et al.*, 2011). GADG is likely synthesized by the promiscuous glycosyltransferase, *agt*, from SAR11 (Figures 3b and c), which dominated the heterotrophic bacterial communities during the mesocosm experiments (Figure 4c).

**Figure 5:**
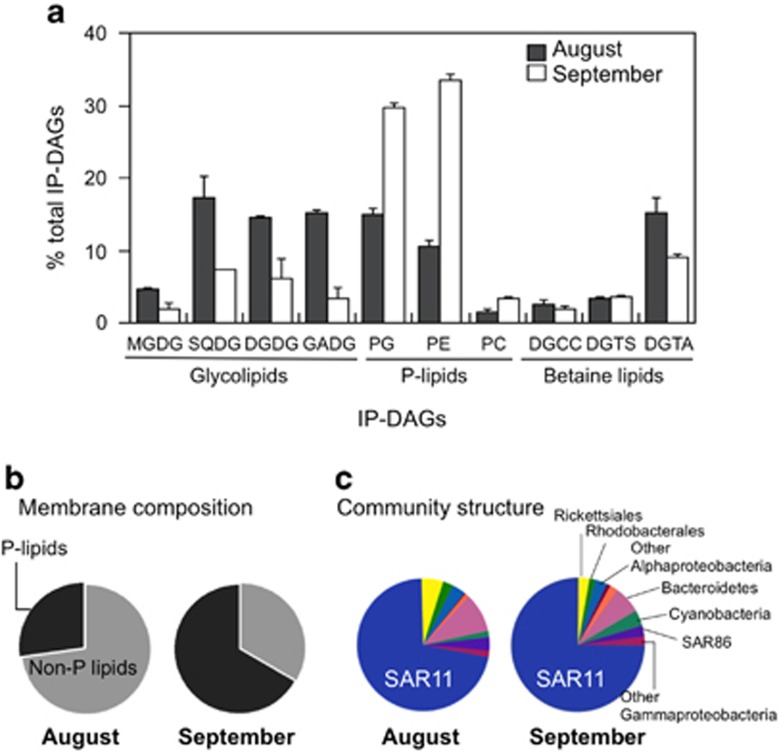
Polar lipids from the heterotrophic bacterial community (0.2–0.8-μm size fraction) at Blanes Bay (North-West Mediterranean) in August and September 2012. (**a**) Contribution of IP-DAGs to membrane composition. (**b**) Percentage of contribution of non-P versus P-lipids. (**c**) Bacterioplankton community structure at the time of sampling (right panel). Abbreviations are as described in Figure 4.

We also analyzed the membrane lipids of heterotrophic bacterial communities in the Western Mediterranean Sea. Samples were collected in August, when P limitation was expected to be severe (Pinhassi *et al.*, 2006), and in September, when P limitation commonly starts to be alleviated. In agreement with our results from the mesocosm experiments, there was a substantial change in membrane lipid composition, despite little change in bacterial community composition between samplings (Figure 5). Non-P lipids represented >70% of the polar lipids in August but only around 30% in September. Non-P lipids in the bacterial fraction were composed of glycolipids and betaine lipids. Among the glycolipids, the sulpholipid, sulphoquinovosyl diacylglycerol (SQDG), diglycosyl diacylglycerol (DGDG) and GADG were the most abundant, although the amount of MGDG was also significantly higher in August. The betaine lipid, diacylglyceryl hydroxymethyl trimethyl-β-alanine (DGTA), was also relatively more abundant in August. These two environmental studies provide direct support for our predictions, based on work with cultured isolates and comparative genomics, that lipid remodelling is a general response by marine heterotrophic bacteria to P deficiency.

## Discussion

### Lipid remodelling is a widespread strategy in marine heterotrophic bacteria under low P conditions

The ability to synthesize non-P lipids seems to be widespread among marine heterotrophic bacteria adapted to low P environments. We found that in P-depleted regions, such as the North Atlantic Subtropical Gyre and the Mediterranean Sea, PlcP may be present in the majority of bacterial cells (Supplementary Figure S1). Although alphaproteobacterial PlcP sequences are the most abundant in marine metagenomes (Figure 2), PlcP appears to be broadly distributed among bacteria with distinct ecologies. Whereas the planktonic oligotrophic SAR11 clade are specialized for the high affinity uptake of small compounds (Giovannoni *et al.*, 2005), PlcP was also identified in the SAR86 clade, believed to dominate the uptake of high molecular weight organic matter (Dupont *et al.*, 2012). Members of groups with a particle-associated lifestyle, such as the *Roseobacter* clade and *Bacteroidetes* (Wagner-Döbler and Biebl, 2006; Fernández-Gómez *et al.*, 2013), also harboured PlcP homologues. Lipid remodelling mediated by PlcP therefore does not appear to be restricted to bacteria with particular lifestyles but rather constitutes part of a generic response to low P among marine bacteria from P-depleted environments.

One previous explanation for the accumulation of non-P lipids in marine heterotrophic bacteria is that they increase cell size without changing the cellular P quota (Thingstad *et al.*, 2005; Van Mooy *et al.*, 2009). However, we observed reductions in the amount of phospholipids per cell without obvious changes in cell size or in estimated cell volume (Supplementary Table S1). Together with the very low membrane P content found in environmental heterotrophic bacteria (Supplementary Table S1), our data suggest an alternative strategy whereby lipid remodelling is used to reduce the cellular P quota.

Enzymes involved in the synthesis of these non-P glycerolipids, including DGTS, SQDG, MGDG and GADG, require DAG as a substrate (Klug and Benning, 2001; Hölzl *et al.*, 2005; Semeniuk *et al.*, 2014). Bacterial lipid synthesis proceeds via a shared phosphatidate (phosphorylated DAG) intermediate (Parsons and Rock, 2013). The generation of DAG requires either the de-phosphorylation of phosphatidate or the removal of the polar head group from a phospholipid by the action of a phospholipase C (for example, PlcP). *Phaeobacter* sp. MED193 as well as the SAR11 representative, HTCC7211, appear to lack homologues of bacterial phosphatidate phosphatases (Icho and Raetz, 1983). Thus it is likely that the major means of generating DAG in marine heterotrophic bacteria is through the action of PlcP (Supplementary Figure S3). This is consistent with the abolition of DGTS synthesis in the *Phaeobacter* sp. MED193 Δ*plcP* deletion mutant (Figure 1h) and the complementation of the *Phaeobacter* sp. MED193 Δ*plcP* deletion mutant with the PlcP of SAR11 strain HTCC7211. On the other hand, in *E*. *coli*, DAG can be generated through the degradation of PG by MdoB (Parsons and Rock, 2013), which explains why glycolipids could accumulate in *E. coli* following overexpression of *agt* from '*Candidatus* Pelagibacter ubique' sp. HTCC7211 without an exogenous supply of DAG. In contrast to the widespread distribution of PlcP homologues across several phyla of marine heterotrophic bacteria, they are largely absent from the genomes of cyanobacteria (Figure 2), which are also capable of lipid remodelling (Van Mooy *et al.*, 2006, 2009). It is likely that cyanobacteria instead generate the required DAG through the action of phosphatidate phosphatases, which are found in their genomes (Nakamura *et al.*, 2007).

The ability to remodel lipids in response to P scarcity appears to be ubiquitous in phytoplankton (Van Mooy *et al.*, 2009), whereas in heterotrophic bacteria it seems to be more restricted to those adapted to low P conditions (Supplementary Figure S1). An explanation for this may be that the typical replacement lipids in cyanobacteria, SQDG and MGDG, are also required for the proper functioning of photosynthetic membranes, for example, in association with photosystem II (Umena *et al.*, 2011). Heterotrophic bacteria, by contrast, appear to require primarily PE and PG as bulk membrane lipids (Parsons and Rock, 2013). There is a selective pressure against the accumulation of genes that confer an insufficient advantage in bacterial genomes (Mira *et al.*, 2001), and this deletional bias has been invoked as an explanation for the patchy distribution of genes conferring an adaptation to P deficiency in the marine environment (Coleman and Chisholm, 2010). An example of this is found among the SAR11 bacteria. '*Candidatus* Pelagibacter ubique' sp. HTCC7211, isolated from the P-deplete Sargasso Sea, contains a higher number and diversity of P acquisition genes than other SAR11 strains (for example, *P. ubiqu*e HTCC1062), thereby increasing its ability to use alternative sources of P (Carini *et al.*, 2014), and is capable of lipid remodelling (Carini *et al.* 2015). In fact, HTCC7211 becomes dominant during the summer months in the Sargasso Sea (Brown *et al.*, 2012), when this region becomes P depleted. In contrast, *P. ubiqu*e HTCC1062, isolated from P-rich waters of the coastal North East Pacific, can only grow on phosphate (Carini *et al.*, 2014), and the lack of *plcP* in its genome suggests that it is not capable of P-lipid remodelling. Thus, it is likely that genes involved in the synthesis of non-P lipids are specifically acquired by marine heterotrophic bacteria as an adaptation to low P conditions. This may explain why non-P lipids in marine heterotrophic bacteria seem to be much more diverse than those in their photosynthetic counterparts.

Whereas sulpholipids are the preferred substitution lipid for cyanobacteria, and betaine lipids for eukaryotic phytoplankton (Van Mooy *et al.*, 2009), heterotrophic bacteria synthesize a broad spectrum of lipids upon P deficiency, including betaine lipids (DGTS and DGTA), and a variety of glycolipids and putative aminolipids (Figures 2). Sulpholipids (SQDG), the glycolipid DGDG and betaine lipids (DGTS, DGTA) have been traditionally ascribed to phytoplankton (Van Mooy and Fredricks, 2010), although production of DGTA in dark seawater incubations using glucose as a precursor has been previously reported (Popendorf *et al.*, 2011), supporting our suggestion of a heterotrophic origin. Furthermore, the gene *sqdB*, involved in SQDG biosynthesis, has recently been detected in environmental heterotrophic bacteria (Villanueva *et al.*, 2014). However, sequences affiliated to heterotrophic bacteria appear to be rare in pelagic marine environments (Van Mooy *et al.*, 2006). Similarly, homologues of glycosyltransferases known to be involved in DGDG synthesis such as *pgt* (Devers *et al.*, 2011; Hölzl *et al.*, 2005) are rare in the marine environment although found in some marine heterotrophic bacteria (for example, *Pelagibaca bermudensis*; Supplementary Table S2, Figure 3a). In our study, only small numbers of cyanobacteria were present in the fractions used for lipid analysis (<3% of total cells), suggesting that the detected lipids are of predominantly heterotrophic origin. How marine heterotrophic bacteria synthesize these lipids, that is, DGTA and DGDG, awaits further characterization.

### The glycosyltransferase Agt is a key enzyme in the synthesis of glycolipids in marine heterotrophic bacteria

We also confirmed that the glycosyltransferase Agt is the enzyme mediating the synthesis of MGDG and GADG in SAR11 bacteria (Figure 3), and report that these lipids are abundant in the membrane lipids of natural bacterial communities in P-deplete waters of the Mediterranean Sea (Figures 4 and 5). To the best of our knowledge, our study constitutes the first report of glucuronic acid lipids in marine environments. Substitution of the acidic phospholipid PG by glucuronic acid-containing glycolipids has also been documented in P-limited cultures of *Brevundimonas diminuta* (formerly *Pseudomonas diminuta*) (Minnikin *et al.*, 1974) and recently in the Sargasso sea SAR11 isolate HTCC7211 (Carini *et al.* 2015). Therefore, it seems that despite the metabolic diversity of the SAR11 clade (Schwalbach *et al.*, 2010, Carini *et al.* 2014), SAR11 isolates from P-depleted systems behave similarly. Both the phospholipid PG and GADG are anionic under physiological conditions, so it may be that they could be interchanged while maintaining the biophysical properties of the membrane. A similar substitution of PG for the anionic SQDG has already been proposed for phytoplankton (Van Mooy *et al.*, 2006). PlcP in *Sinorhizobium meliloti* only displayed activity towards the zwitterionic phospholipids PE and phosphatidylcholine (Zavaleta-Pastor *et al.*, 2010) but the substrate specificity of SAR11 PlcP, as well as environmental PlcP homologues, warrants further investigation.

### Physiological consequences of lipid remodelling

Despite the variety of surrogate non-P lipids, marine heterotrophic bacteria nonetheless seem to revert to phospholipid-dominated membranes under P sufficiency, similar to what has been observed in phytoplankton (Martin *et al.*, 2011). Yet a study in yeast has shown that DGTS can functionally substitute for the phospholipid phosphatidylcholine without an apparent phenotype in culture (Riekhof *et al.*, 2014). It is therefore puzzling why heterotrophic bacteria do not simply maintain a constant low P membrane composition. In plants, phospholipids have been proposed as P storage molecules, which can be mobilized upon P deficiency (Tjellström *et al.*, 2008). Indeed, we observed that heterotrophic bacterial communities in the Mediterranean Sea accumulated phospholipids upon relief of P limitation (Figures 4 and 5), while there was no major change in community composition, suggesting that excess P is stored in phospholipids. This is consistent with the use of phospholipids as a P reservoir: on return to P limitation, PlcP-mediated degradation of membrane phospholipids might result in a net release of P for diversion to other cellular uses. An additional explanation for the prevalence of phospholipids under P sufficiency is that membrane proteins appear to have evolved in a phospholipid-dominated environment. For example, activity of certain membrane transporters can be enhanced by specific interactions with phospholipids (Laganowsky *et al.*, 2014).

In summary, our results demonstrate that the ability to substitute phospholipids for non-P lipids under P deprivation is a common strategy in marine heterotrophic bacterial communities adapted to low P environments. Central to the remodelling process is a phospholipase, PlcP, which is widespread in marine surface waters where phosphorus is scarce. Our data point to lipid remodelling as an important ecological adaptation enabling natural heterotrophic bacteria to thrive in low P marine environments.
