# Supplementary material for: Lipid remodelling is a widespread strategy in marine heterotrophic bacteria upon phosphorus deficiency
Source: ISME J. 2015 Nov 13;10(4):968–78. doi: 10.1038/ismej.2015.172 (PMC4796936; doi:10.1038/ismej.2015.172)
Supplement: Supplementary Information [file ismej2015172x1.doc]

**SUPPLEMENTARY INFORMATION**

**Lipid remodelling is a widespread strategy in marine heterotrophic bacteria upon phosphorus deficiency**

Marta Sebastián^1*^, Alastair F. Smith^2*^, José M. González^3^, Helen F. Fredricks^4^, Benjamin Van Mooy^4^, Michal Koblížek^5^, Joost Brandsma^6^, Grielof Koster^6^, Mireia Mestre^1^, Behzad Mostajir^7^, Paraskevi Pitta^8^, Anthony D. Postle^6^, Pablo Sánchez^1^, Josep M. Gasol^1^, David J Scanlan^2^, Yin Chen^2^

This file contains:

**Supplementary Materials and Methods**

**Supplementary Table 1.** Phosphorus content in the membrane lipids of marine bacteria.

**Supplementary Table 2.** Putative genes involved in phosphorus-free lipid synthesis in the genomes of PlcP-containing marine bacterial isolates.

**Supplementary Table 3.** Oligonucleotide primers used in the RT-PCR experiments.

**Supplementary Table 4.** Bacterial strains and plasmids used for molecular genetic work in this study.

**Supplementary Table 5.** Oligonucleotide primers used for molecular genetic work in this study.

**Supplementary Figure 1.** Distribution of PlcP homologs in the Global Ocean Survey and *Tara* Oceans databases.

**Supplementary Figure 2.** Taxonomic affiliation of PlcP homologs retrieved from Marine Metatranscriptomic databases

**Supplementary Figure 3.** Proposed pathway of synthesis of non-phosphorus lipids through phospholipids and PlcP in marine heterotrophic bacteria.

**Supplementary Figure 4.** Glucuronic acid diacylglycerol (GADG) detected in the membranes of marine heterotrophic bacteria in the Western Mediterranean Sea, August 2012.

**References**

**Supplementary Materials and Methods:**

**P starvation experiments: time course of alkaline phosphatase activity, expression of PlcP in marine isolates and membrane lipid analyses**

*Phaeobacter* sp. MED193 and *Dokdonia* sp. MED134 were grown in 50 mL of Marine Broth diluted 1:20 with filtered seawater supplemented with 10 mM glucose, 5 mM NH_4_Cl, 200 µM K_2_HPO_4_, 1µM Fe and 1ml/L vitamin solution (2 mg biotin, 2 mg folic acid, 10 mg pyridoxine HCl, 5 mg riboflavin, 5 mg thiamine, 5 mg nicotinic acid, 0.1 mg cyanocobalamin, 5 mg *p*-aminobenzoic acid in 100 mL of distilled water, pH: 7). Cells were harvested by centrifugation at the onset of stationary phase. Half of the cells were re-suspended in P-replete medium (+P, 200 µM K_2_HPO_4_) and the other half in medium without added phosphate (-P). Samples were monitored for P starvation by performing alkaline phosphatase activity assays. Experiments were performed in duplicate. Twenty hours after inoculation samples for RNA were harvested and P was added back to the –P cultures. Samples for RNA were collected again 2 h after P addition. RNA samples (25 ml) were centrifuged for 10 min at 12,000 ×g and the pellets were immediately frozen in liquid nitrogen and stored at -80 °C. RNA was extracted using TRI reagent (SIGMA) and treated with Turbo DNase (Ambion). RNA was reverse transcribed using random hexamers and the SuperScriptIII kit (Invitrogen) according to the manufacturer's instructions. PCR was performed using primers designed to amplify internal fragments of *PlcP* and rplU (ribosomal protein L21) of both strains and also for the alkaline phosphatase gene *phoX* in *Phaeobacter* sp. MED193 (Supplementary Table 3). rplU served as a control for cDNA synthesis. One µl of cDNA were used as template in the following reaction: 1 cycle of 94 °C 5 min, 35 cycles of 94 °C 30s, 50 °C 30s, 72 °C 1 min, and 1 cycle of 72 °C 10 min. PCR products were sequenced to confirm the gene of interest. Alkaline phosphatase activity was determined in triplicate subsamples by monitoring the rate of hydrolysis of the fluorogenic substrate 6,8-difluoro-4-methylumbelliferyl phosphate (DifMUP, Invitrogen, Eugene, OR, USA) at a final concentration of 10 µM.

The *Erythrobacter* sp. NAP1 cultures were grown in an artificial sea water medium as described previously (Koblíže*k et a*l., 2003). The phosphate replete medium contained 0.3 mM NaH_2_PO_4_ and 1 mM glutamic acid as a sole source of organic carbon. To induce phosphorus-limited conditions the culture medium was reformulated to contain 10 mM glutamic acid and 10 µM NaH_2_PO_4_. The cells were grown in Erlenmeyer flasks on an orbital shaker (120 RPM) to assure proper aeration. Illumination was provided by a bank of luminescent tubes at an irradiance level of 150 µmol *quanta* m^-2^ s^-1^ in a 12:12 hour light–dark cycle. The cultures were grown up to stationary phase and cells were collected for RNA. RNA extraction and the RT-PCR experiments were performed as described above but using the 16S rRNA gene as control for cDNA synthesis. For the lipid analyses, cells were grown in triplicate as described above and 48 h after inoculation 4 mL aliquots were filtered onto a 0.2 µm pore size durapore membrane. Membrane lipids from these cultures were extracted and quantified as described elsewhere (Popendor*f et a*l., 2013).

The biovolume of the bacterial cells was analyzed as described in Massan*a et a*l., (2009).

**Construction and complementation of the *plcP* mutant in *Phaeobacter* sp. MED193**

Flanking regions to the 5’ and 3’ ends of MED193_17359 were amplified using primers designed with *Hin*dIII and *Bam*HI restriction sites on the external and internal primers, respectively. Marker exchange mutants were then constructed as described in Lidbur*y et a*l., (2014), with the modification that transconjugants were selected using glycine betaine as a sole nitrogen source.

Complementation of the *plcP* mutant was achieved by amplifying MED193_17359 plus the 400 base pairs upstream of that gene using primers with *Hin*dIII and *Bam*HI sites engineered into the 5’ and 3’ primers, respectively. This was subcloned into pGEM-T (Promega) before ligation into the broad host range plasmid pBBR1MCS-Km(Kovac*h et a*l., 1995). The plasmid was then introduced into Δ*plcP* as described in Lidbur*y et a*l., (2014). Complementation using the *plcP* homolog from SAR11 strain HTCC7211 was achieved by chemically synthesizing the SAR11 gene (locus tag, PB7211_983) fused at the 5’ end to the 400 base pair upstream promoter sequence from MED193 (carried out by Genscript, NJ, USA). Complementation then proceeded as described above, using *Hin*dIII and *Bam*HI sites synthesized at the 5’ and 3’ ends, respectively to subclone the construct into pGEM-T. All bacterial strains and plasmids used for genetic work are shown in Supplementary Table 4, and the primers in Supplementary Table 5.

**Characterization of the *plcP* deletion mutant of *Phaeobacter* sp. MED193**

*Phaeobacter* sp. MED193 strains were grown in PCR-S11 medium (Rippk*a et a*l., 2000) modified by the addition of 10 mM glucose and 1 mL/L vitamin solution. After initial growth to late exponential phase, cells were pelleted by centrifugation at 9,000 x *g* for 5 minutes. Cell pellets were resuspended in the same volume of either PCR-S11 medium with added P (50 μM) or with no added P. The cultures were tracked for 4 days following resuspension. On each day, cell density was measured using the optical density at 540 nm (OD_540_) and a 5 mL aliquot of culture was collected for lipid analysis. Cells were pelleted by centrifugation, then resuspended in 0.9 mL 20 mM ammonium acetate and internal standard containing 25 nmol 17:0/17:0 PC (Avanti Polar Lipids, Alabaster, AL.) in 1:1 methanol:dichloromethane (DCM). Lipids were extracted following a modified Bligh-Dyer procedure (Bligh and Dyer, 1959), the solvent removed under nitrogen and the dried lipids stored at -80 °C.

Prior to analysis, dried extracts were resuspended in 1:1 methanol:DCM. liquid chromatography-mass spectrometry analysis employed a Dionex 3400RS HPLC system coupled to an AmazonSL quadrupole ion trap (Bruker Scientific) via an electrospray ionisation interface. Separation was on a 150 mm Nucleosphere HILIC column (Macherey-Nagel) at 30 °C, with a flow rate of 150 μL min^-1^. Samples were run on a gradient of 95% acetonitrile to 28% 10 mM ammonium acetate, with 2 and 5 minute holds at the start and end of each run, respectively. Ionisation conditions were an end cap voltage of 4,500 V, 8 L min^-1^ drying gas at 250 °C and a nebulising gas pressure of 1 psi. Selected masses were targeted for fragmentation to MS2, using the SmartFrag functionality of the Bruker TrapControl software to select an appropriate voltage. Masses selected for fragmentation were those identified as corresponding to DGTS (738.7 and 764.7) and the PC internal standard (762.7). The relative abundance of DGTS was expressed as the ratio of the sum of the peak areas for DGTS to the peak area for the PC internal standard, normalized to the OD_540_ of the culture.

**Phylogeny of PlcP and PlcP homologs in metagenomics and metatranscriptomic databases**

Phylogenetic analysis were performed using 225 PlcP-like sequences from marine bacterial isolates (Integrated Microbial Genomes) and 1129 sequences of environmental PlcP homologs obtained from the GOS database (Yoosep*h et a*l., 2008). These sequences were retrieved through BLASTP searches using *Phaeobacter* sp. MED193 PlcP (MED193_17359) as a query. An e-value <10^-40^ was used as the cut off value. The metagenome sequences were organised into 243 clusters using the CD-HIT program (Li and Godzik, 2006) and applying a 90% similarity threshold. The amino-acid sequences were aligned using MUSCLE (Edgar and Edgar, 2004). A maximum likelihood tree was generated with the R software package phangorn using the JTT model. Confidence estimates for the internal branches were obtained using 100 bootstrap replicates. Normalization of PlcP reads in the metagenome was achieved by performing a BLASTP search using RecA, an essential single copy gene, from *Phaeobacter* sp. MED193 (MED193_04366). Again, an e-value <10^-40^ was used as a cut-off. To correct for differences in sequence length of PlcP (265 a.a.) and RecA (354 a.a.), the number of PlcP hits was divided by the ratio of the lengths of PlcP and RecA.

For the analyses of PlcP in the *Tara* Oceans metagenomes we first performed tBLASTn searches using *Phaeobacter* sp. MED193 PlcP (MED193_17359) as a query against the non-redundant Ocean Microbial Reference Gene Catalog (OM-RGC; Sunagawa et al. 2015). The OM-RGC IDs positive for the PlcP gene were then subsetted from the *Tara* Oceans gene profile table (normalized counts of each non-redundant OM-RGC gene across *Tara* Oceans stations), which can be downloaded from <http://ocean-microbiome.embl.de/companion.html>. PlcP homologues were then grouped according to ecologically relevant taxonomic groups. This table was used to obtain the total counts of PlcP genes for each taxonomic group, and their abundance across all stations. Only surface water samples (5 m) were taken into account for these analyses. PlcP counts were divided by RecA counts, obtained as described for PlcP but using *Phaeobacter* sp. MED193 RecA as query.

PlcP homologs in metatranscriptomic databases were identified by tBLASTn using *Phaeobacter* sp. MED193 (MED193_17359) as query, with an e-value <0.001. This subset of reads was aligned against NCBI-nr database using BLASTX to confirm their homology to PlcP and their putative taxonomic affiliation. For this analysis we used those metatranscriptomic databases from oligotrophic marine systems that are publicly available (Ottese*n et a*l., 2013, 2014; Vila-Cost*a et a*l., 2010).

**Characterization of SAR11 glycosyltransferase**

The *atg* homolog from *Ca.* P. ubique HTCC7211 (locus_tag, PB7211_960, Genbank accession number, EDZ60637) was codon optimized for *E. coli* and chemically synthesized by Genscript (NJ, USA) with restriction sites engineered at each end (Supplementary Table S5). The construct was cloned into the pET28a expression vector (Novagen) and transformed in *E. coli* BLR(DE3). Cultures were grown at 37 °C in M9 medium to an OD_600_ of around 0.6 before induction with 0.2 mM IPTG and incubation overnight at 16 °C. Lipids extracts were analyzed by the LC-MS method described above. Masses selected for fragmentation were 774.6 and 788.6, which preliminary studies had indicated corresponded to ammoniated 34:1 MGDG and GADG, respectively. Chromatograms were scanned for neutral losses of 177 or 193, corresponding to the loss of hexosyl or hexuronosyl groups, respectively. Glycolipid abundance was expressed as the ratio of the peak area of the neutral loss fragment to that of the PC internal standard.

**Screening of the genomes and metagenomic scaffolds for the presence of Pho-boxes**

The prediction of potential PhoB binding sites within the genomes and environmental scaffolds was based on the method described in Yua*n et a*l., (2006). A position-weight matrix was constructed using 34 Pho-box sequences known for *Sinorhizobium meliloti* and 10 Pho-box sequences known for *Escherichia coli* (Yua*n et a*l., 2006). This matrix was later used to scan the intergenic region of the genomes for the highest score (log-odds), using an in-house Python script. Based on previous knowledge of genes that are known to be up-regulated under P stress we established a threshold that divided high-scoring Pho boxes (>8) from low scoring-Pho boxes. Only high-scoring Pho boxes are shown in Fig. 3. This prediction tool worked well with *Proteobacteria*, except for SAR11 and related strains due to their high AT-content.

**Collection of samples for Intact Polar lipids (IP-DAGs) in the environment**

*Mesocosm study*

A transportable floating mesocosms platform (Mostaji*r et a*l., 2013) was deployed in September 2011 in one of the south-easternmost basins of the Mediterranean, the Cretan Sea, in the framework of the MESOAQUA European project. Mesocosms of ~16 m^3^ were filled with natural seawater from the surrounding environment. Two mesocosms were enriched with phosphate (100 nM), and 2 served as controls. Samples for IP-DAGs were taken on days 0, and 6 of the experiment. Two litres were filtered on precombusted 0.2 µm pore size alumina membranes for total community lipids, and 4-L were prefiltered through 0.8 µm pore size and collected on precombusted 0.2 µm pore size alumina membranes for the lipids of the heterotrophic bacterial community. The bacterial community composition was evaluated by catalyzed reporter deposition fluorescence *in situ* hybridization as described elsewhere (Sebastiá*n et a*l., 2012). Flow cytometry was also used to confirm that the 0.2-0.8 µm size fraction did not contain a significant number of cyanobacterial cells, following the protocol described elsewhere (Ferrer*a et a*l., 2011).

*Blanes Bay Microbial Observatory*

The Blanes Bay Microbial Observatory is an oligotrophic coastal station located in the North-Western Mediterranean Sea (<http://www.icm.csic.es/bio/projects/icmicrobis/bbmo/>). Samples for lipids were taken in August and September 2012. Two litres of seawater were filtered on a 0.2 µm pore size durapore filter for total community lipids, and 4-L were pre-filtered through 0.8 µm pore size and collected on a 0.2 µm pore size durapore filter. To confirm that the 0.2 – 0.8 µm size-fraction was composed almost exclusively of heterotrophic bacteria, we examined the community composition of this fraction by pyrosequencing of bacterial 16S rRNA genes. To this end, 10 L samples were sequentially filtered through 0.8 and 0.2 µm pore size filters. The filters were stored at -80 °C and DNA was extracted as described in Massan*a et a*l., (1997). Hypervariable V1-V3 16S rRNA gene regions were amplified by PCR and 454 GS FLX+ pyrosequenced using primers 28F/519R. Reads from 150 to 600 bp were quality checked (Phred quality average >25) by using a 50 bp sliding window in QIIME(Caporas*o et a*l., 2010). Pyrosequencing errors were reduced with the Denoiser in QIIME. Reads were clustered into OTUs with a 97% similarity threshold with UCLUST in QIIME. Chimeras were removed with ChimeraSlayer (Haa*s et a*l., 2011), with SILVA108 as a reference database, in Mothur (Schlos*s et a*l., 2009). Taxonomy assignment was done using SILVA Incremental Aligner (SINA v1.2.11). Samples were randomly normalized at the minimum sequencing depth for comparative purposes. Flow cytometry was also used to confirm that the 0.2-0.8 µm size fraction did not contain a significant number of cyanobacterial cells, representing only 3% of the total bacterial cells counts.

|  | treatment | **10^6^ P atoms cell^-1^** | **Biovolume (μm^3^)** |
| --- | --- | --- | --- |
| ***Phaeobacter* sp. MED193** | P-replete | 0.54 (0.19) | 0.075 (n=520) |
|  | P-deplete | 0.27 (0.15) | 0.077 (n=494) |
| ***Dokdonia* sp. MED134** | P-replete | 2.63 (1.0) | 0.105 (n=560) |
|  | P-deplete | 1.72 (0.28) | 0.082 (n=499) |
| ***Synechococcus* WH8102** | P-deplete | 0.16***** | N.A |
| ***Pelagibacter ubique* HTCC1062** | P-replete | 1.5***** | N.A |
| **Eastern Mediterranean**  **Sea Heterotrophic bacteria** | P-replete | 0.25-0.30 | N.A |
|  | P-deplete | 0.052-0.13 | N.A |

**Table S1**. Phosphorus content in the membrane lipids of marine bacteria. This value was estimated as the sum of P atoms contained in the membrane phospholipids. Data are presented as the mean of three replicates (and standard deviation) or as a range when only two replicates were available. The biovolume of the cells was estimated by means of image analysis. Numbers in parenthesis indicate the number of cells used for the analyses.

* Van Mooy, et al. (2009) Phytoplankton in the ocean use non-phosphorus lipids in response to phosphorus scarcity. *Nature* 458 (7234):69–72.

| **Table S2.** Putative genes involved in phosphorus-free lipid synthesis in the genomes of PlcP-containing marine isolates. The genomes of all marine isolates in the IMG database were BLASTP searched using MED193_17359 as a query (e-value < 1e-20) in order to construct a database of PlcP-containing marine isolates. To find genes in the vicinity of PlcP, the nucleotide sequence 5 kb up- and down-stream of PlcP was extracted. tBLASTn searches were performed against these sequences using a 1e-20 E-value cut-off. To detect putative non-P synthesis genes in the genomes of these isolates we used BLASTP searches with a 1e-20 E-value cut-off. Query sequences used: Agt - Agau_C200037; BtaB - Q93TQ0_RHOSH; OlsF - Spro_2569; SqdB - Q9L8S7_RHIML; Pgt - mlr5650 | | | | |
| --- | --- | --- | --- | --- |
|  | **non-P lipid synthesis genes in the vicinity of PlcP** | **Presence of non-P lipid synthesis genes in the genome, and putative non-P lipid synthesized** | | |
| **taxon_name** | **Neighbour** | **MGDG/GADG** | **DGTS** | **SQDG** |
| *Hoeflea phototrophica* DFL-43 | BtaBA | Agt | BtaB |  |
| *Phaeobacter daeponensis* TF-218, DSM 23529 (scaffold version) | BtaBA |  | BtaB |  |
| *Phaeobacter* sp. MED193 | BtaBA |  | BtaB |  |
| *Sagittula stellata* E-37 | BtaBA |  | BtaB | SqdB |
| *Planctomyces maris* DSM 8797 | BtaBA |  |  |  |
| *Rhodopirellula baltica* SH 1 | BtaBA |  |  |  |
| *Rhodopirellula baltica* SH28 | BtaBA |  |  |  |
| *Rhodopirellula baltica* SWK14 | BtaBA |  |  |  |
| *Rhodopirellula baltica* WH47 | BtaBA |  |  |  |
| *Saprospira grandis* HR1, DSM 2844 | BtaBA |  |  |  |
| *Saprospira grandis* Lewin | BtaBA |  |  |  |
| alpha proteobacterium SCGC AAA536-G10 | Agt |  |  |  |
| alpha proteobacterium SCGC AAA536-K22 | Agt |  |  |  |
| alpha proteobacterium sp. HIMB59 | Agt |  |  |  |
| *Amorphus coralli* DSM 19760 | Agt |  |  | SqdB |
| *Aurantimonas coralicida* DSM 14790 | Agt |  |  |  |
| *Aurantimonas manganoxydans* SI85-9A1 | Agt |  |  |  |
| beta proteobacterium NB0016 | Agt |  |  |  |
| Candidatus *Pelagibacter* sp. HTCC7211 | Agt |  |  |  |
| *Caulobacter crescentus* CB15 | Agt |  |  |  |
| *Caulobacter crescentus* NA1000 | Agt |  |  |  |
| *Citromicrobium bathyomarinum* JL354 | Agt |  | BtaB |  |
| *Citromicrobium* sp. JLT1363 | Agt |  | BtaB |  |
| *Cucumibacter marinus* DSM 18995 | Agt |  |  | SqdB |
| *Desulfobulbus mediterraneus* DSM 13871 | Agt |  |  |  |
| *Erythrobacter litoralis* HTCC2594 | Agt |  |  |  |
| *Erythrobacter* sp. NAP1 | Agt |  | BtaB |  |
| *Erythrobacter* sp. SD-21 | Agt |  |  |  |
| *Fulvimarina pelagi* HTCC2506 | Agt |  |  |  |
| gamma proteobacterium sp. HTCC5015 | Agt |  |  |  |
| *Kordiimonas gwangyangensis* DSM 19435 | Agt |  |  |  |
| *Labrenzia aggregata* IAM 12614 | Agt |  | BtaB | SqdB |
| *Labrenzia alexandrii* DFL-11 | Agt |  | BtaB | SqdB |
| *Labrenzia* sp. DG1229 | Agt |  | BtaB | SqdB |
| *Leucothrix mucor* DSM 2157 | Agt |  | BtaB |  |
| *Limnobacter* sp. MED105 | Agt |  |  |  |
| *Loktanella vestfoldensis* SKA53 | Agt |  | BtaB |  |
| marine bacterium Betaproteobacteria HIMB624 | Agt |  |  |  |
| *Maritalea myrionectae* DSM 19524 | Agt |  |  |  |
| *Methylophaga aminisulfidivorans* MP, KCTC 12909 | Agt |  |  |  |
| *Methylophaga frappieri* JAM7 | Agt |  |  |  |
| *Methylophaga nitratireducenticrescens* JAM1 | Agt |  |  |  |
| *Nisaea denitrificans* DSM 18348 | Agt |  | BtaB |  |
| *Nisaea* sp BAL199 | Agt |  | BtaB |  |
| *Nitrobacter* sp. Nb-311A | Agt |  |  |  |
| *Pelagibacterium halotolerans* B2 | Agt |  |  | SqdB |
| *Polynucleobacter necessarius asymbioticus* QLW-P1DMWA-1 | Agt |  |  |  |
| *Pseudomonas aeruginosa* WC55 | Agt |  |  |  |
| *Pseudovibrio* sp. JE062 | Agt |  | BtaB | SqdB |
| *Pusillimonas* sp. T7-7 | Agt |  |  |  |
| *Roseibium* sp. TrichSKD4 | Agt |  | BtaB |  |
| *Rubritalea marina* DSM 17716 | Agt |  |  |  |
| *Sphingomonas* sp. KC8 | Agt |  |  |  |
| *Sphingomonas* sp. S17 | Agt |  |  |  |
| *Sphingomonas* sp. SKA58 | Agt |  | BtaB |  |
| *Sphingopyxis alaskensis* RB2256 | Agt |  |  |  |
| *Sphingopyxis baekryungensis* DSM 16222 | Agt |  | BtaB |  |
| *Stappia stellulata* DSM 5886 | Agt |  | BtaB |  |
| *Terasakiella pusilla* DSM 6293 | Agt |  |  |  |
| *Thalassobaculum salexigens* DSM 19539 | Agt |  | BtaB |  |
| *Thalassospira lucentensis* DSM 14000 | Agt |  |  | SqdB |
| *Thalassospira profundimaris* WP0211 | Agt |  |  |  |
| *Thalassospira xiamenensis* M-5, DSM 17429 | Agt |  |  |  |
| *Thiomicrospira crunogena* XCL-2 | Agt |  |  |  |
| *Thiomicrospira kuenenii* DSM 12350 | Agt |  |  |  |
| *Verrucomicrobia* bacterium SCGC AAA300-K03 | Agt |  |  |  |
| *Citreicella* sp. 357 | Pgt |  |  |  |
| *Citreicella* sp. SE45 | Pgt |  |  | SqdB |
| *Pelagibaca bermudensis* HTCC2601 | Pgt |  |  |  |
| *Rhodobacter sphaeroides* KD131 | Pgt |  | BtaB | SqdB |
| *Algicola sagamiensis* DSM 14643 | OlsF |  |  |  |
| *Alteromonas macleodii* AltDE1 | OlsF |  |  |  |
| *Alteromonas macleodii* ATCC 27126 | OlsF |  |  |  |
| *Alteromonas macleodii* Balearic Sea AD45 | OlsF |  |  |  |
| *Alteromonas macleodii* Black Sea 11 | OlsF |  |  |  |
| *Alteromonas macleodii* Deep ecotype, DSM 17117 | OlsF |  |  |  |
| *Alteromonas* sp. S89 | OlsF |  |  | SqdB |
| *Catenovulum agarivorans* YM01 | OlsF |  |  |  |
| *Enterovibrio calviensis* DSM 14347 | OlsF |  |  |  |
| gamma proteobacterium BDW918 | OlsF |  |  | SqdB |
| gamma proteobacterium IMCC1989 | OlsF |  |  |  |
| gamma proteobacterium IMCC3088 | OlsF |  |  |  |
| *Gayadomonas joobiniege* G7 | OlsF |  |  |  |
| *Glaciecola agarilytica* 4H-3-7+YE-5 | OlsF |  |  |  |
| *Haliea rubra* CM41_15a, DSM 19751 | OlsF |  |  |  |
| *Melitea salexigens* DSM 19753 | OlsF |  |  |  |
| *Microbulbifer variabilis* ATCC 700307 | OlsF | Agt |  | SqdB |
| *Pseudoalteromonas tunicata* D2 | OlsF |  |  |  |
| *Rheinheimera baltica* DSM 14885 | OlsF |  |  |  |
| *Shewanella algae* ACDC | OlsF |  |  |  |
| *Shewanella baltica* BA175 | OlsF |  |  |  |
| *Shewanella baltica* OS117 | OlsF |  |  |  |
| *Shewanella baltica* OS155 | OlsF |  |  |  |
| *Shewanella baltica* OS183 | OlsF |  |  |  |
| *Shewanella baltica* OS185 | OlsF |  |  |  |
| *Shewanella baltica* OS195 | OlsF |  |  |  |
| *Shewanella baltica* OS223 | OlsF |  |  |  |
| *Shewanella baltica* OS625 | OlsF |  |  |  |
| *Shewanella frigidimarina* NCIMB 400 | OlsF |  |  |  |
| *Shewanella* *piezotolerans* WP3 | OlsF |  |  |  |
| *Shewanella* sp. HN-41 | OlsF |  |  |  |
| *Shewanella* sp. MR-4 | OlsF |  |  |  |
| *Shewanella* sp. MR-7 | OlsF |  |  |  |
| *Shewanella* sp. W3-18-1 | OlsF |  |  |  |
| *Shewanella violacea* DSS12 | OlsF |  |  |  |
| *Spongiibacter tropicus* DSM 19543 | OlsF |  |  |  |
| *Stenotrophomonas* sp. SKA14 | OlsF | Agt |  |  |
| *Cytophaga hutchinsonii* ATCC 33406 | Uncharacterised glycosyltransferase |  |  |  |
| *Eudoraea adriatica* DSM 19308 | Uncharacterised glycosyltransferase |  |  |  |
| Flavobacteriaceae bacterium S85 | Uncharacterised glycosyltransferase |  |  |  |
| *Flavobacterium* sp. SCGC AAA536-P05 | Uncharacterised glycosyltransferase |  |  |  |
| *Fulvivirga imtechensis* AK7 | Uncharacterised glycosyltransferase |  |  |  |
| *Gracilimonas tropica* DSM 19535 | Uncharacterised glycosyltransferase | Agt |  |  |
| *Microscilla marina* ATCC 23134 | Uncharacterised glycosyltransferase |  |  |  |
| *Owenweeksia hongkongensis* DSM 17368 | Uncharacterised glycosyltransferase |  |  |  |
| *Pedobacter* sp. BAL39 | Uncharacterised glycosyltransferase |  |  |  |
| *Polaribacter* sp. MED152 | Uncharacterised glycosyltransferase |  |  |  |
| *Prolixibacter bellariivorans* ATCC BAA-1284 | Uncharacterised glycosyltransferase |  |  |  |
| *Saccharicrinis fermentans* DSM 9555 | Uncharacterised glycosyltransferase |  |  |  |
| *Tenacibaculum ovolyticum* DSM 18103 | Uncharacterised glycosyltransferase |  |  |  |
| *Caminibacter mediatlanticus* TB-2 | Uncharacterised glycosyltransferase |  |  |  |
| *Blastopirellula marina* SH 106T, DSM 3645 | Uncharacterised glycosyltransferase |  |  |  |
| *Algoriphagus mannitolivorans* DSM 15301 | Uncharacterised glycosyltransferase |  |  |  |
| *Algoriphagus marincola* DSM 16067 | Uncharacterised glycosyltransferase |  |  |  |
| *Algoriphagus* sp. PR1 | Uncharacterised glycosyltransferase |  |  |  |
| *Algoriphagus vanfongensis* DSM 17529 | Uncharacterised glycosyltransferase |  |  |  |
| *Aquiflexum balticum* BA160, DSM 16537 | Uncharacterised glycosyltransferase |  |  |  |
| *Belliella baltica* BA134, DSM 15883 | Uncharacterised glycosyltransferase |  |  |  |
| *Cyclobacterium marinum* Raj, DSM 745 | Uncharacterised glycosyltransferase |  |  |  |
| *Echinicola pacifica* DSM 19836 | Uncharacterised glycosyltransferase |  |  |  |
| *Echinicola vietnamensis* KMM 6221, DSM 17526 | Uncharacterised glycosyltransferase |  |  |  |
| *Rhodonellum psychrophilum* DSM 17998 | Uncharacterised glycosyltransferase |  |  |  |
| *Rhodonellum psychrophilum* GCM71, DSM 17998 (NZ_Draft) | Uncharacterised glycosyltransferase |  |  |  |
| *Rhodothermus marinus* SG0.5JP17-171 | Uncharacterised glycosyltransferase | Agt |  |  |
| *Rhodothermus marinus* SG0.5JP17-172 | Uncharacterised glycosyltransferase | Agt |  |  |
| *Croceibacter atlanticus* HTCC2559 | Uncharacterised glycosyltransferase |  |  |  |
| *Dokdonia* sp. MED134 | Uncharacterised glycosyltransferase |  |  |  |
| *Flavobacteria* bacterium BBFL7 | Uncharacterised glycosyltransferase |  |  |  |
| *Joostella marina* En5, DSM 19592 | Uncharacterised glycosyltransferase |  |  |  |
| *Kordia algicida* OT-1 | Uncharacterised glycosyltransferase |  |  |  |
| *Mesonia mobilis* DSM 19841 | Uncharacterised glycosyltransferase |  |  |  |
| *Robiginitalea biformata* HTCC2501 | Uncharacterised glycosyltransferase |  |  |  |
| *Mesoflavibacter zeaxanthinifaciens* DSM 18436 | Uncharacterised glycosyltransferase |  |  |  |
| *Aliagarivorans marinus* DSM 23064 | - |  |  |  |
| *Aliagarivorans taiwanensis* DSM 22990 | - |  |  |  |
| alpha proteobacterium SCGC AAA536-B06 | - | Agt |  |  |
| *Alteromonas* sp. SN2 | - |  |  |  |
| *Amphritea japonica* ATCC BAA-1530 | - |  |  |  |
| *Aquimarina latercula* DSM 2041 | - |  |  |  |
| *Aquimarina muelleri* DSM 19832 | - |  |  |  |
| *Arcobacter* sp. CAB | - |  |  |  |
| *Arcobacter* sp. L | - |  |  |  |
| *Cellulophaga algicola* IC166, DSM 14237 | - |  |  |  |
| *Coraliomargarita akajimensis* DSM 45221 | - | Agt |  |  |
| *Dokdonia* sp. MED134 | - |  |  |  |
| *Ensifer meliloti* AK83, DSM 23913 | - |  |  | SqdB |
| *Ensifer meliloti* CIAM1775 | - |  | BtaB | SqdB |
| *Flavobacterium frigidarium* DSM 17623 | - |  |  |  |
| gamma proteobacterium IMCC2047 | - |  |  |  |
| gamma proteobacterium SCGC AAA076-P09 | - |  |  |  |
| gamma proteobacterium SCGC AAA076-P13 | - |  |  |  |
| *Gilvimarinus chinensis* DSM 19667 | - |  |  | SqdB |
| *Hirschia baltica* ATCC 49814 | - | Agt |  |  |
| *Hirschia maritima* DSM 19733 | - | Agt |  |  |
| *Idiomarina baltica* OS145 | - |  |  |  |
| *Idiomarina loihiensis* L2TR | - |  |  |  |
| *Idiomarina sediminum* DSM 21906 | - |  |  |  |
| *Leeuwenhoekiella blandensis* MED217 | - |  |  |  |
| *Maribacter antarcticus* DSM 21422 | - |  |  |  |
| *Marinobacter adhaerens* HP15, DSM 23420 | - | Agt |  |  |
| *Marinobacter algicola* DG893 | - | Agt |  |  |
| *Marinobacter aquaeolei* VT8 | - | Agt |  |  |
| *Marinobacter daepoensis* DSM 16072 | - | Agt |  |  |
| *Marinobacter hydrocarbonoclasticus* ATCC 49840 | - | Agt |  |  |
| *Marinobacter manganoxydans* MnI7-9 | - | Agt |  |  |
| *Marinomonas mediterranea* MMB-1, ATCC 700492 | - |  |  |  |
| *Marinomonas posidonica* IVIA-Po-181 | - |  |  |  |
| *Marinomonas* sp. MWYL1 | - |  |  |  |
| *Marinomonas ushuaiensis* DSM 15871 | - |  |  |  |
| *Mesoflavibacter zeaxanthinifaciens* S86 | - |  |  |  |
| *Methylomonas methanica* MC09 | - |  |  |  |
| *Methylophaga thiooxydans* DMS010 | - |  |  |  |
| *Muricauda ruestringensis* B1, DSM 13258 | - |  |  |  |
| *Nitratireductor aquibiodomus* RA22 (Draft1) | - |  | BtaB | SqdB |
| *Nitratireductor indicus* C115 | - |  | BtaB | SqdB |
| *Nitrosococcus halophilus* Nc4 | - | Agt |  |  |
| *Photobacterium angustum* S14 | - |  |  |  |
| *Photobacterium leiognathi mandapamensis* svers.1.1 | - |  |  |  |
| *Photobacterium* sp. SKA34 | - |  |  |  |
| *Pseudoalteromonas arctica* A 37-1-2 | - |  |  |  |
| *Pseudoalteromonas citrea* NCIMB 1889 | - |  |  |  |
| *Pseudoalteromonas flavipulchra* 2ta6 (Draft assembly 1) | - |  |  |  |
| *Pseudoalteromonas haloplanktis* ANT/505 | - |  |  |  |
| *Pseudoalteromonas luteoviolacea* 2ta16 (Draft assembly 1) | - |  |  |  |
| *Pseudoalteromonas marina* mano4 | - |  |  |  |
| *Pseudoalteromonas piscicida* ATCC 15057 | - |  |  |  |
| *Pseudoalteromonas piscicida* JCM 20779 | - |  |  |  |
| *Pseudoalteromonas rubra* ATCC 29570 | - |  |  |  |
| *Pseudoalteromonas* sp. TW-7 | - |  |  |  |
| *Pseudoalteromonas spongiae* UST010723-006 | - |  |  |  |
| *Pseudomonas putida* CSV86 | - | Agt |  |  |
| *Reinekea blandensis* MED297 | - |  |  |  |
| *Salinisphaera shabanensis* E1L3A | - | Agt |  | SqdB |
| *Salisaeta longa* DSM 21114 | - | Agt |  |  |
| SAR86 cluster bacterium SAR86C | - |  |  |  |
| *Shewanella waksmanii* ATCC BAA-643 | - |  |  | SqdB |
| *Simiduia agarivorans* DSM 21679 | - |  |  |  |
| *Simiduia agarivorans* SA1 | - |  |  |  |
| *Sulfurospirillum arcachonense* DSM 9755 | - |  |  |  |
| *Synechococcus* sp. RCC 307 | - | Agt |  | SqdB |
| *Thiocapsa marina* 5811, DSM 5653 | - | Agt |  |  |
| *Thiorhodococcus drewsii* AZ1 | - | Agt |  | SqdB |
| *Thiorhodovibrio* sp. 970 | - | Agt |  |  |
| *Verrucomicrobiales* sp. DG1235 | - | Agt |  |  |
| *Vibrio nigripulchritudo* ATCC 27043 | - |  |  |  |
| *Zunongwangia profunda* SM-A87 | - |  |  |  |

**Table S3.** Oligonucleotide primers used in the RT-PCR experiments.*

| **Gene** | **Strain** | **Locus_tag** | **Forward primer** | **Reverse primer** | **Amplicon Size (bp)** |
| --- | --- | --- | --- | --- | --- |
| *plcP* | *Phaeobacter* sp. MED193  *Dokdonia* sp*.* MED134  *Erythrobacter* sp. NAP1 | MED193_17359  MED134_03774  NAP1_12828 | 5'-GGCGATATCGTTGATGCCTGG-3'  5'-CAYGAYGARAWRCTKCGWAAA-3'  5'-TTCTTCTTGAGACGCCACCC-3' | 5'-TGGATGTGACCGCAGATCACC-3'  5'-TGWATRTGWCCRCARAYYACA-3'  5'-AATGTGGCAAATCTCCCCGT-3' | 505  340  220 |
| *phoX* | *Phaeobacter* sp. MED193 | MED193_05784 | 5’-GARGAGAACWTCCACGGYTA-3' | 5’-GATCTCGATGATRTGRCCRAAG-3' | 600 |
| *rplU* | *Phaeobacter* sp. MED193  *Dokdonia* sp*.* MED134 | MED193_03872  MED134_01280 | 5'-GACTGGCGGCAAGCAGTACAAA-3'  5'-GTAGAGATAGCAGGGCAGCA-3' | 5'-ATCTACCAAAGGAGCACCAAC-3'  5'-CTATAGCYGGGGCGCCTAAAGT-3' | 150  150 |
| 16S rRNA | *Eubacteria universal primer* (515F, 805R) | 16S rRNA gene | 5'-CCTACGGGAGGCAGCAG-3' | 5'-CCGTCAATTCMTTTGAGTTT-3' | 290 |

*All primers, except from 515F/805R (Caporas*o et a*l., 2011), are designed in this study.

Table S4. Bacterial strains and plasmids used for molecular genetic work in this study.

| **Plasmid/strain** | **Description/use** | **Source** |
| --- | --- | --- |
| *Phaeobacter* sp. MED193 | Wild type. | Muthusam*y et a*l., 2014 |
| *Phaeobacter* sp. MED193 Δ*plcP* | *Phaeobacter* sp. MED193 with *med193_17359* disrupted. | This study |
| *Phaeobacter* sp. MED193 Δ*plcP^MED193^* | Δ*plcP* mutant complemented with pBBR1plcP. | This study |
| *E. coli* JM109 | Host for cloning. | Promega |
| *E. coli* S17.1 | Electrocompetent cells. Used for conjugation. | Lab collection |
| *E. coli* BLR(DE3) *pLysS* | Heterologous protein expression. | Promega |
| p34S-Gm | Source of gentamycin resistance gene cassette (Gm^R^). | Dennis and Zylstra, 1998 |
| pGEM-T Easy | Cloning vector. | Promega |
| pK18*mobsacB* | Suicide vector for maker exchange mutagenesis in *Phaeobacter* sp. MED193 | Schäfe*r et a*l., 1994 |
| pBBR1MCS-Km | Broad-host-range plasmid. | Kovac*h et a*l., 1995 |
| pET28a | Heterologous protein expression. | Merck Bioscience |
| pK18plcP | *plcP* of MED193, together with its native promoter, cloned into pBBR1MCS-Km using the 17359_prom primer pair. | This study |
| pBBR1plcP | Fragment cloned into pBBR1MCS-Km from MED193 using the 17359_prom primer pair. | This study |
| pUC57agt | PB7211_960 (*agt* homologue) codon optimized for *E. coli* and cloned into pUC57 with NdeI and BamHI restriction sites at the 5’ and 3’ ends, respectively. | Genscript Corporation |
| pUC57plcP | PB7211_983 (*plcP* homologue) plus the promoter sequence from MED193_17359, cloned into pUC57 with *Hin*dIII and *Bam*HI restriction sites at the 5’ and 3’ ends, respectively. | Genscript Corporation |

Table S5 Oligonucleotide primers used for molecular genetic work in this study. Restriction sites are underlined.

| **Locus Tag** | **Use** | **Forward primer (5**'**-3**'**)** | **Reverse primer (5**'**-3**'**)** |
| --- | --- | --- | --- |
| MED193_17359 | Δ*plcP* construction (upstream region). | GTCTAAGCTTTGAGGATGACGACGATGTTC | CTATGGATCCGGTGTCCGCTTCGTGACTAT |
| MED193_17359 | Δ*plcP* construction (downstream region). | CTATGGATCCTTGTCGAGCGAGACAATGG | ATCTAAGCTTCGCTCATATAGGGGGAGGTT |
| MED193_17359 | Confirmation of Δ*plcP.* | AGCCATTTTTCACCACCAAG | CCCAGAACCCCGTAGTGATA |
| MED193_17359 (plus promoter) | Δ*plcP^MED193^* construction. | AGTCAAGCTTAACTGGTCAGCAAGCCAACT | AGTCGGATCCCATCGGGTAGATCCCCTATACA |
| PB7211_960 | Confirmation of SAR11 *agt* (codon optimized for *E. coli*). | ATCCGCAAGTCAATGGTGTT | GTCACGTTTCACCGGATTTT |

**a)**

**b)**

Supplementary Figure 1 Distribution of PlcP homologs in marine metagenomes. Sampling sites are coloured according to the estimated percentage of cells with a PlcP homolog. a) Distribution in the Global Ocean Sampling database. BLASTP searches were performed using *Phaeobacter* sp. MED193 PlcP (MED193_17359) as query (e-value < 10^-40^). The number of reads at each site was normalized using BLASTP hits to RecA from MED193 (MED193_04366; e-value < 10^-40^). b) Distribution of PlcP in the *Tara* dataset. The relative abundance of PlcP relative to RecA across all stations was calculated using the table of abundances of eggNOG families obtained from the *Tara* OCEANS-Global Ocean Microbiome Information and Data resource (see Supplementary Methods for further details).

n= 367

Verrucomicrobia

unknown


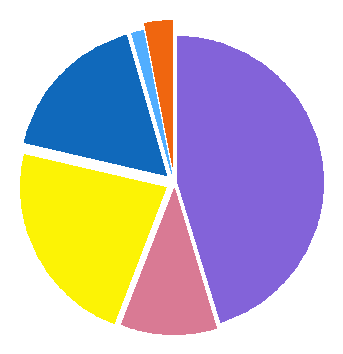


Alphaproteobacteria

Bacteroidetes

Betaproteobacteria

Gammaproteobacteria

**Supplementary Figure 2.** Taxonomic affiliation of PlcP homologs retrieved from Marine Metatranscriptomic databases. PlcP homologs were identified by tBLASTn using *Phaeobacter* sp. MED193 (MED193_17359) as query with an e-value <0,001. This subset of reads was back aligned against the NCBI-nr database using BLASTx to confirm their homology to PlcP and their putative taxonomic affiliation.

**Supplementary Figure 3.** Proposed pathway of synthesis of non-phosphorus lipids through phospholipids and PlcP in marine heterotrophic bacteria. Diacylglycerol (DAG) is released by PlcP from phospholipids which serves as a substrate by a variety of pathways. Three such pathways are shown here: the synthesis of the betaine lipid, diacylglyceryltrimethylhomoserine (DGTS) by BtaB and BtaA, the synthesis of the glycolipids monoglucosyldiacylglycerol (MGDG) and glucuronic acid diacylglycerol (GADG) by Agt, and the synthesis of sulfolipid sulfoquinovosyl diacylglycerol (SQDG) by SqdB, SqdC and SqdD.

**
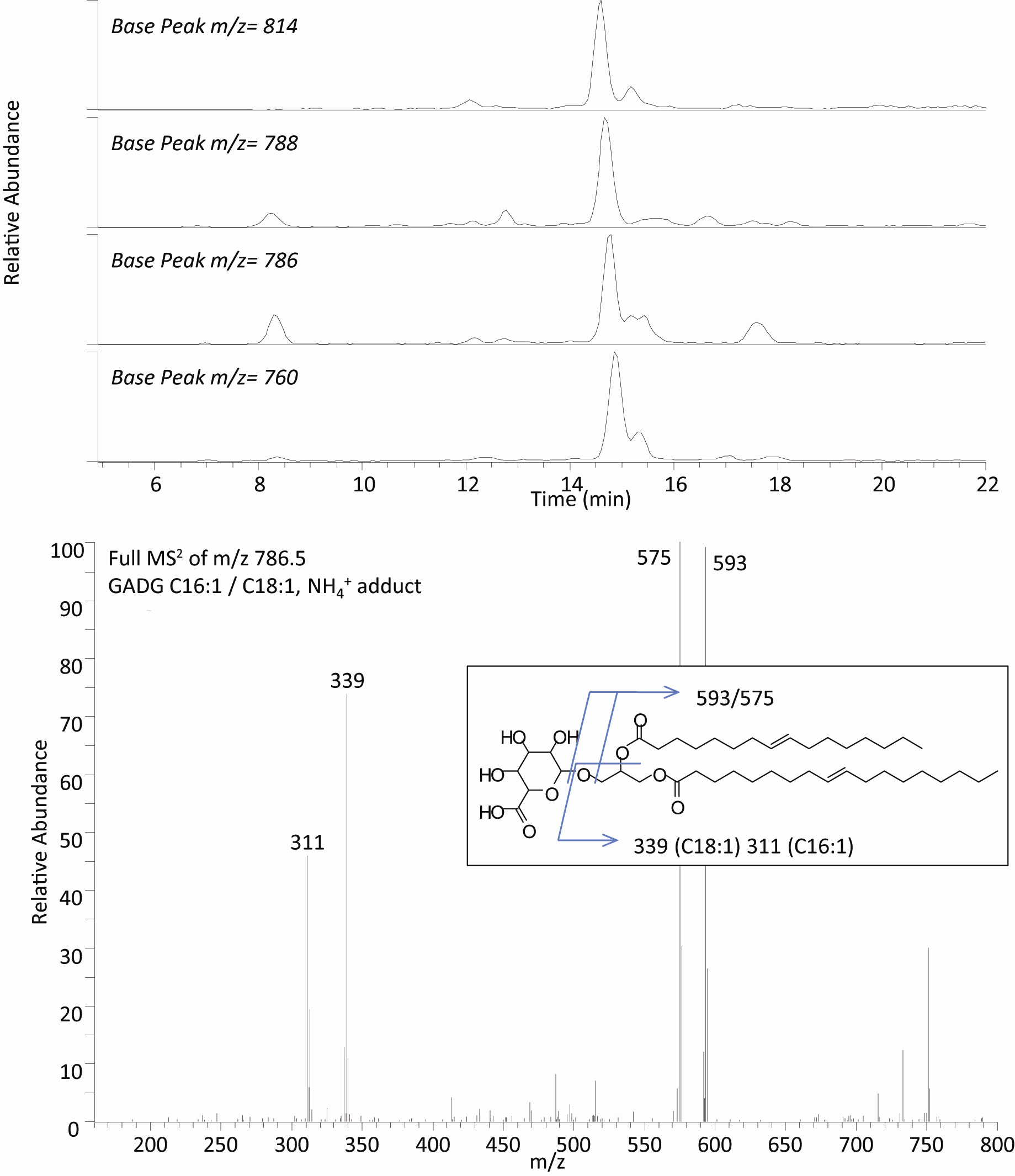
**

**Supplementary Figure 4.** Glucuronic acid diacylglycerol (GADG) detected in the membranes of marine heterotrophic bacteria in the Western Mediterranean Sea, August 2012. Base peak chromatograms show a distinct group of relatively polar molecules eluting at 14.7 minutes. The MS2 spectrum of m/z 786.5 shows the head group neutral losses of m/z 193 and 211, which are evidenced by the fragments of m/z 593 and 575. The fragments of m/z 311 and 339 are indicative of 18:1 and 16:1 fatty acid moieties. Positions of the double bonds in the fatty acid moieties are arbitrary. The exact conformation of the hexose group is unknown.

**References**

Caporaso JG, Kuczynski J, Stombaugh J, Bittinger K, Bushman FD, Costello EK, *et al.* (2010). QIIME allows analysis of high-throughput community sequencing data. *Nat Methods* **7**:335–336.

Caporaso JG, Lauber CL, Walters WA, Berg-Lyons D, Lozupone CA, Turnbaugh PJ, *et al.* (2011). Global patterns of 16S rRNA diversity at a depth of millions of sequences per sample. *Proc Natl Acad Sci USA* **108 Suppl** :4516–22.

Dennis JJ, Zylstra GJ. (1998). Plasposons: modular self-cloning minitransposon derivatives for rapid genetic analysis of gram-negative bacterial genomes. *Appl Environ Microbiol* **64**:2710–5.

Edgar RC, Edgar RC. (2004). MUSCLE: multiple sequence alignment with high accuracy and high throughput. *Nucleic Acids Res* **32**:1792–7.

Ferrera I, Gasol JM, Sebastián M, Hojerová E, Koblízek M. (2011). Comparison of growth rates of aerobic anoxygenic phototrophic bacteria and other bacterioplankton groups in coastal Mediterranean waters. *Appl Environ Microbiol* **77**:7451–8.

Haas BJ, Gevers D, Earl AM, Feldgarden M, Ward D V., Giannoukos G, *et al.* (2011). Chimeric 16S rRNA sequence formation and detection in Sanger and 454-pyrosequenced PCR amplicons. *Genome Res* **21**:494–504.

Koblížek M, Béjà O, Bidigare RR, Christensen S, Benitez-Nelson B, Vetriani C, *et al.* (2003). Isolation and characterization of *Erythrobacter* sp. strains from the upper ocean. *Arch Microbiol* **180**:327–338.

Kovach ME, Elzer PH, Hill DS, Robertson GT, Farris MA, Roop RM, *et al.* (1995). Four new derivatives of the broad-host-range cloning vector pBBR1MCS, carrying different antibiotic-resistance cassettes. *Gene* **166**:175–176.

Li W, Godzik A. (2006). Cd-hit: A fast program for clustering and comparing large sets of protein or nucleotide sequences. *Bioinformatics* **22**:1658–1659.

Lidbury I, Murrell JC, Chen Y. (2014). Trimethylamine N-oxide metabolism by abundant marine heterotrophic bacteria. *Proc Natl Acad Sci USA* **111**:2710–5.

Massana R, Murray AE, Preston CM, DeLong EF. (1997). Vertical distribution and phylogenetic characterization of marine planktonic Archaea in the Santa Barbara Channel. *Appl Environ Microbiol* **63**:50–56.

Massana R, Unrein F, Rodríguez-Martínez R, Forn I, Lefort T, Pinhassi J, *et al.* (2009). Grazing rates and functional diversity of uncultured heterotrophic flagellates. *ISME J* **3**:588–596.

Mostajir B, Le Floc’h E, Mas S, Pete R, Parin D, Nouguier J, *et al.* (2013). A new transportable floating mesocosm platform with autonomous sensors for real-time data acquisition and transmission for studying the pelagic food web functioning. *Limnol Oceanogr Methods* **11**:394–409.

Muthusamy S, Baltar F, Gonzalez JM, Pinhassi J. (2014). Dynamics of metabolic activities and gene expression in the *Roseobacter* clade bacterium *Phaeobacter* sp. strain MED193 during growth with thiosulfate. *Appl Environ Microbiol* **80**:6933–6942.

Ottesen E a, Young CR, Eppley JM, Ryan JP, Chavez FP, Scholin C a, *et al.* (2013). Pattern and synchrony of gene expression among sympatric marine microbial populations. *Proc Natl Acad Sci USA* **110**:E488–97.

Ottesen E a, Young CR, Gifford SM, Eppley JM, Marin R, Schuster SC, *et al.* (2014). Ocean microbes. Multispecies diel transcriptional oscillations in open ocean heterotrophic bacterial assemblages. *Science* **345**:207–12.

Popendorf KJ, Fredricks HF, Van Mooy BAS. (2013). Molecular ion-independent quantification of polar glycerolipid classes in marine plankton using triple quadrupole MS. *Lipids* **48**:185–195.

Rippka R, Coursin T, Hess W, Lichtle C, Scanlan DJ, Palinska KA, *et al.* (2000). *Prochlorococcus marinus* Chisholm *et al*. 1992 subsp. *pastoris* subsp. nov. strain PCC 9511, the first axenic chlorophyll *a_2_*/*b_2_*-containing cyanobacterium (Oxyphotobacteria). *Int J Syst Evol Microbiol* **50**:1833–1847.

Schäfer a, Tauch a, Jäger W, Kalinowski J, Thierbach G, Pühler a. (1994). Small mobilizable multi-purpose cloning vectors derived from the *Escherichia coli* plasmids pK18 and pK19: selection of defined deletions in the chromosome of *Corynebacterium glutamicum*. *Gene* **145**:69–73.

Schloss PD, Westcott SL, Ryabin T, Hall JR, Hartmann M, Hollister EB, *et al.* (2009). Introducing mothur: Open-source, platform-independent, community-supported software for describing and comparing microbial communities. *Appl Environ Microbiol* **75**:7537–7541.

Sebastián M, Pitta P, González JM, Thingstad TF, Gasol JM. (2012). Bacterioplankton groups involved in the uptake of phosphate and dissolved organic phosphorus in a mesocosm experiment with P-starved Mediterranean waters. *Environ Microbiol* **14**:2334–47.

Vila-Costa M, Rinta-Kanto JM, Sun S, Sharma S, Poretsky R, Moran MA. (2010). Transcriptomic analysis of a marine bacterial community enriched with dimethylsulfoniopropionate. *ISME J* **4**:1410–1420.

Yooseph S, Li W, Sutton G. (2008). Gene identification and protein classification in microbial metagenomic sequence data via incremental clustering. *BMC Bioinformatics* **9**:182.

Yuan Z-C, Zaheer R, Morton R, Finan TM. (2006). Genome prediction of PhoB regulated promoters in *Sinorhizobium meliloti* and twelve proteobacteria. *Nucleic Acids Res* **34**:2686–97.

.
